# Consensus on relevant psychosocial interventions applied in health institutions to prevent psychological violence at work: Delphi method

**DOI:** 10.1186/s13104-023-06680-w

**Published:** 2024-01-05

**Authors:** Luis Fidel Abregú-Tueros, Cinthia Jannete Bravo-Esquivel, Sheyla Karol Abregú-Arroyo, Roger Dos Santos-Rosa, José Luis Galve-Manzano

**Affiliations:** 1 Research Institute in Psychology, University of San Martín de Porres, Lima, Peru; 2 Faculty of Medicine, Universidad Peruana Cayetano Heredia, Lima, Peru; 3 Servicio de Laboratorio, Hospital Nacional EsSalud “Ramiro Prialé Prialé”, Huancayo, Peru; 4 Faculty of Medicine, Federal University of Rio Grande do Sul, Porto Alegre, Brazil; 5 CAERD-Collective for Applied Educational Research and Development, Guadalajara, Spain

**Keywords:** Psychological violence at work, Prevention, Consensus of interventions, Health professionals, Delphi, Peru

## Abstract

**Objective:**

Studies on psychological violence in the workplace (PVW) in Latin America have focused on incidence values. In contrast, studies on preventive interventions (PIs) in the health sector are very limited. Our objective was to determine to what extent there is consensus on the most relevant characteristics of the psychosocial interventions applied in the prevention of PVW in health institutions in Peru. To that end, health professionals with knowledge and experience in PVW at the national level were recruited, and the Delphi consensus technique was applied.

**Results:**

The consensus study was developed in four stages that included three phases of Delphi consultation. In the third consultation phase, 428 experts participated in 25 analysis groups from 66 health institutions in the country. A total of 70.3% of the participants were women, and 27.6% of the participants worked in nursing and emergency services. After the Delphi consensus analysis, we obtained a list of 10 hierarchical psychosocial interventions to prevent PVW in the country. Most notable were interventions based on the prior resolution of interprofessional conflicts, on the visibility of incidents to generate an inverse effect and on experiential training to improve assertive and empathic communication skills.

**Supplementary Information:**

The online version contains supplementary material available at 10.1186/s13104-023-06680-w.

## Introduction

Psychological violence in the workplace (PVW) is defined as aggressive behaviour towards workers during their professional practice, either physically or psychologically (e.g., verbally, with threats and/or with some type of humiliation) [1]. PVW among health care professionals can be generated by superiors or colleagues but also by patients and/or visitors [2]. PVW is related to interpersonal conflicts and motivational demands [3, 4]. These two factors are exacerbated in the health sector because psychosocial risk factors are common in health care services, when patients experience long wait times and differences between expectations and the services received [3, 5–7].

In addition to emotional exhaustion [8], the negative consequences of PVW among healthcare professionals include decreased quality of care and job satisfaction and increased emotional stress. These factors negatively influence organizational commitment [7, 9].

Workplace violence of healthcare professionals in the USA reached 61.3% of the total [10], and in 22 Latin American countries they identified 66.7% of cases [11, 12]. Psychological violence in healthcare professionals in Chile was 39.1% [13], and in nurses in Colombia 38.7% of cases [14]. In Peru, there are 186 076 health care professionals representing 46.1% of the total number of health sector workers: 403 848 workers [15], and of this number of health care professionals, it is estimated that internal psychological violence affects about 36.2% of professionals [16].

From a gender perspective in the health sector, the factor that exacerbates PVW is the invisibilisation of women's work and the occupational segregation of women, despite the fact that the majority of nurses with overloaded roles are women [17], while medical positions are mostly filled by male staff. In Peru there are more than 80% women in nursing and about 45% in medicine [17]. The gender approach in public health policies requires preventive approaches and strategies on PVW to strengthen equity and resilience in the health workforce [18, 19].

Since the promulgation of international guidelines to address PVW in the health sector [20], researchers have studied strategies to prevent or reduce such violence [21], exploring alternative prevention methods [22], such as psychosocial interventions (PIs) in interprofessional settings [23] within specific contexts [24].

PIs in the health sector in developed countries have been documented through systematic reviews [1, 4, 6, 7, 25–28], mainly aimed at preventing PVW between nurses or nurses and doctors. There are few studies on interprofessional care groups [8, 21, 22, 29]. In the field of PVW prevention in the health sector, programs with long-term effectiveness are scarce [1], and it is unknown whether PIs prevent or reduce PVW in interprofessional groups that include administrative personnel [21, 30]. The PIs to prevent PVW are very limited, both in Latin America and Peru. Therefore, the present study is important because: (a) it will contribute to reduce the incidence of psychological violence reported for Peru [16]; (b) to apply psychosocial interventions in the country's health institutions to promote interprofessional integration; and (c) to propose policies for the prevention of psychosocial health at work that will contribute to increase job satisfaction and improve the organisational commitment of healthcare professionals. For this reason, several authors [24, 31–34] have pointed out the need to obtain expert opinions to identify the most effective interventions through consensus.

With this initial report, we want to guide the prevention of PVW in health institutions in the country. The objective of this study was to come to a consensus on the PIs that have been applied effectively to prevent PVW in the country's health institutions. To this end, health professionals with knowledge and experience in PVW at the national level were recruited, and the Delphi consensus technique was applied.

## Main text

### Methods

We used the mixed Delphi technique, whose advantages include anonymity and controlled feedback. This technique allows the gathering of participants from different geographical areas, in our case, professionals with common work experience and knowledge about PVW in the Peruvian health sector, to facilitate consensus [31].

**Fig. 1:**
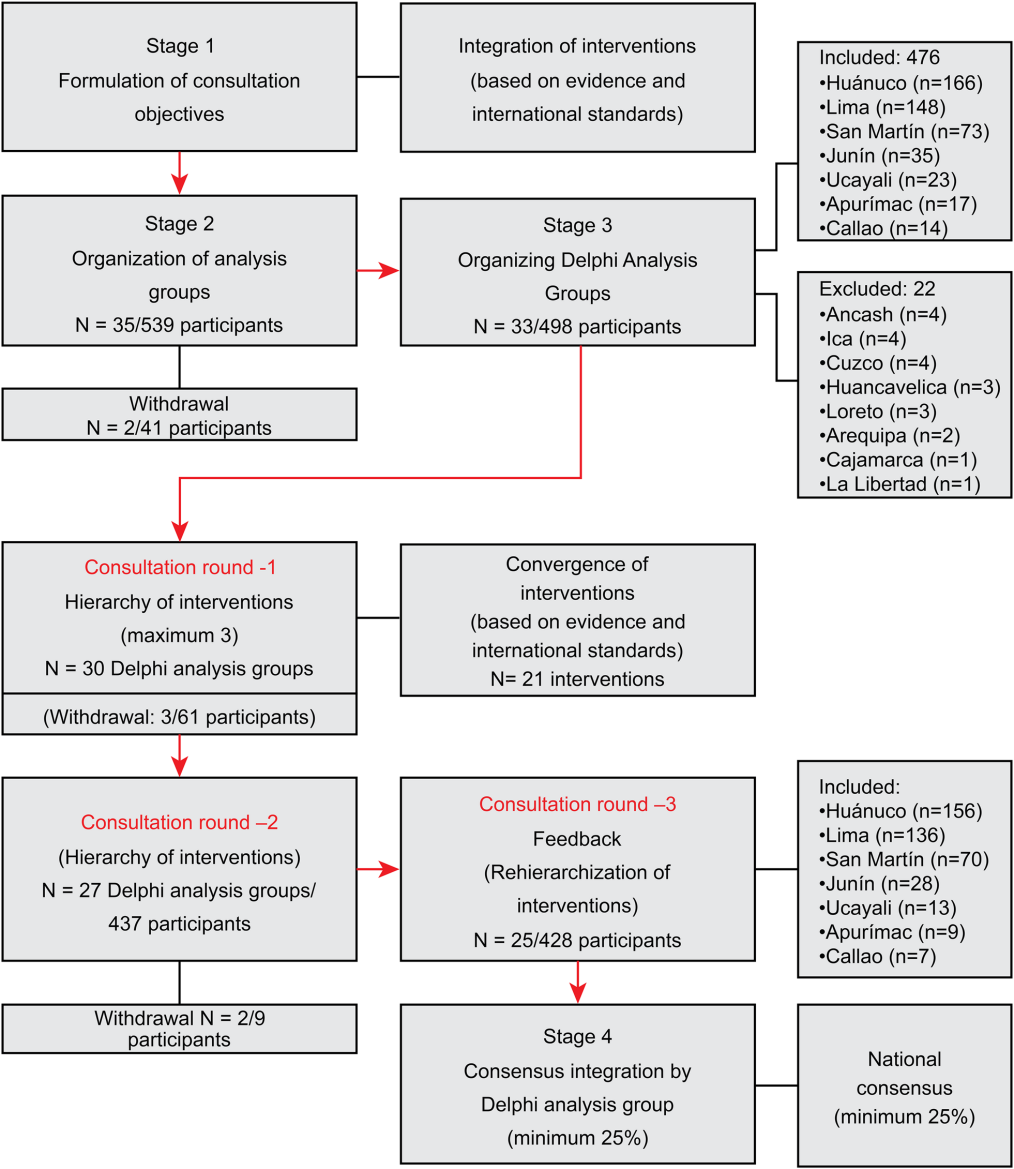
Delphi consensus process

The Delphi consensus process was developed in four stages between August 2021 and February 2023. The first three stages were online and the last was in person. The three rounds of Delphi consultation correspond to the third stage of analysis. The third round of consultation was face to face (October 2022 to February 2023) (Fig. 1).

In the first stage, 30 experts from the emergency and admission services of five public hospitals in the city of Lima were invited to participate and informed about the scope of the study, the hierarchical process and the consensus. They were then asked to integrate a list with PIs to prevent PVW based on evidence [1, 4, 7, 25, 26] and international regulations [20]. At this stage (August 2021), a list of 21 interventions was drawn up to serve as a frame of reference for the subsequent Delphi consultation phases (Fig. 1).

In the second stage (September and December 2021), 35 analysis groups with an equal number of coordinators were organized, and national health professionals and administrative personnel knowledgeable about PVW and strategies for prevention and mediation were identified. Inclusion criteria for participants were: (a) health professional or administrative staff in the health sector of both sexes; (b) with experience and knowledge of PVW; (c) with more than three months of work experience; (d) from any of the three regions of the country; (e) voluntary participation until the 3rd Delphi consultation round. Exclusion criteria were: (a) staff with no experience or knowledge of PVW, (b) probationary period (up to three months). The selection criteria for health centres were: (a) located in any of the three regions of the country; (b) having six or more experts (Table 1; Additional file 2).

**Table 1:** Socio-labour characteristics of the studied population

Characteristics	Total (%) n = 148	p*	Sex	
			Male (%)	Female (%)
Provenance				
Coastal Region	142 (33.2)	0.001	43 (30.3)	99 (69.7)
Sierra Region	65 (15.2)		13 (20.0)	52 (80.0)
Jungle Region	221 (51.6)		71 (32.1)	150 (67.9)
Service				
Nurcing	61 (14.3)	0.001	13 (21.3)	48 (78.7)
Emergencies	57 (13.3)		19 (33.3	38 (66.7)
Obstetrics	33 (7.7)		4 (12.1)	29 (87.9)
Medicine	31 (7.2)		16 (51.6)	15 (48.4)
Laboratory and biochemistry	26 (6.1)		8 (30.8)	18 (69.2)
Other services (n = 33)	184 (43.0)		45 (24.4)	139 (75.6)
Administration	36 (8.4)		20 (55.6)	16 (44.4)
Rotation to another service				
None	223 (52.1)	0.001	69 (30.9)	154 (69.1)
One to two times	166 (38.8)		48 (28.9)	118 (71.1)
Three to four times	37 (8.6)		10 (27.0)	27 (73.0)
Five or mor times	2 (0.5)		0 (0.0)	2 (100.0)

^*^In *X*^2^ test for *p* < *0.05*

In the third stage, the Delphi analysis groups were reduced to 33 groups to maintain the required minimum of six experts in each group [35, 36]. A total of eight analysis groups were excluded. In the first round of Delphi consultation, each expert was asked to propose up to three interventions to prevent PVW. A list of convergent interventions was then organized using the 21 of the suggested interventions. The second round of consultations was developed with 27 analysis groups that ranked the 21 interventions, from 1 for the most important or effective intervention to 21 for the least important intervention. In the third round of Delphi consultation, after feedback on the results of the second round, a new hierarchy of interventions was requested, and repetitive hierarchies in the same hierarchy were eliminated (Fig. 1).

In the fourth round of consultations, the consensus and discrepancies for each intervention were integrated by each Delphi analysis group, and then the interventions were ranked according to their relative frequency. Next, a hierarchy of consensus and discrepancies was established at the national level. The criterion to consider consensus by analysis group for the integrated consensus at the national level was a minimum of 25% [31, 36].

### Statistical analysis

The consensus hierarchy was determined [31] by calculating the relative frequencies of the 21 reference interventions. To determine the proportionality of the sample size according to socio-occupational and geographical characteristics, the chi-square test was applied.

The datasets generated and analysed during the current study [37] are available in the [Figshare] repository. [Persistent web link to datasets]. DOI [https://doi.org/10.6084/m9.figshare.23255270.v1].

### Results

#### Description of participants

Until the second round of consultation, 569 health professionals and administrative personnel from the health sector participated at the national level. In the third and last round of Delphi consultation, 428 experts from 66 health institutions in seven departments of the country (Huánuco, Lima, San Martín, Junín, Ucayali, Apurímac, and El Callao) participated, of which 51.6% came from the Jungle region. A total of 70.3% of participants were women, most of whom worked in the obstetrics and nursing services in the Sierra region; and had rotated from their placements three to five times a year (Table 1). A total of 14.3% of the participants worked in the nursing service and 13.3% in the emergency service. They had been employed for four months to 35 years.

More than half (52.1%) of the experts had not been rotated from their services in the last year. According to the X^2^ test, the sample was heterogeneous in terms of origin, services, service rotation, and sex (Table 1).

#### Consensus on psychosocial interventions

After reranking following feedback on the results of the second round of Delphi consultation, there was consensus that the condition for success of psychosocial interventions to prevent PVW in the country's health institutions is emotional support and solidarity between the interprofessional team and the administrative staff (Table 2). It was noted that this support is more important than institutional support. This support is complemented by PIs focused on the timely and adequate resolution of interpersonal conflicts by competent officials or private consultants (Table 2; Additional file 1).

**Table 2:** Consensus and hierarchical discrepancy of preventive interventions

Code	Preventive intervention	Consensus		Discrepancy	
		Hierarchy	%^a^	Hierarchy	%
PI01	Through emotional support and solidarity by colleagues	1°	50.5		
PI02	Through support and solidarity by bosses or officials	2°	38.1		
PI11	Immediately solving interpersonal conflicts by bosses or supervisors	3°	36.3		
PI13	Encouraging the fearless reporting of psychological violence	4°	32.2		
PI16	Through training on assertive and empathetic communication skills	5°	30.4		
PI14	Through awareness raising to report psychological violence	6°	30.1		
PI10	Through immediate actions for the prevention and punishment of psychological violence	7°	29.0		
PI17	Through social activities to improve interpersonal relationships between workers and bosses or supervisors	8°	27.3		
PI05	Through preventive awareness programs	9°	25.1		
PI03	With greater presence of security personnel	10°	24.8		
PI20	Through staff meetings to prevent harassment and intimidation at work			1°	80.8
PI06	Through training for officials and workers on the prevention of violence at work			2°	79.4
PI15	Developing follow-up to staff with bullying behaviours			3°	79.0
PI08	Through legal support for complaints to the authorities			4°	78.7
PI19	By promoting preventive institutional policies			5°	78.5
PI04	With the support of the workers' unions			6°	78.5
PI21	With institutional support in guidance and specialized counselling			7°	78.3
PI12	With assessment of the risk of psychological violence at work			8°	77.6
PI07	With timely institutional support during incidents			9°	77.3
PI09	With psychological accompaniment to victims of psychological violence			10°	76.6
PI18	By promoting procedures for the prevention and recovery of psychological violence			11°	76.4

^a^Minimum 25% (25 Delphi analysis groups: n = 6–37 participants)

According to the consultation, the PIs located between the fourth and sixth hierarchy to prevent PVW involve the implementation of PVW visibility programs through appropriate communication channels and integration with other interventions (e.g., PI oriented to the development of PVW skills), assertive and empathetic communication and awareness of how to report PVW cases (Table 2).

Other PIs considered important were the application of administrative and/or judicial sanctions for those who committed PVW (consensus: 29%). In the eighth hierarchy, we find PIs with experiential activities aimed at improving interpersonal relationships among health professionals, administrative personnel and officials. In the ninth hierarchy are the PIs based on the development of awareness programs to reduce PVW. The tenth hierarchy concerns PIs that help prevent PVW (consensus: 24.8%), such as the presence of security personnel in work environments (Table 2; Additional file 1).

According to gender, the most demanded consensuses were in females for the psychosocial interventions PI16, PI13, PI17 and PI11 whose percentage variations compared to males are: PV = 14.5%; PV = 8.5%, PV = 4.9%; PV = 3.1% respectively (Table 2). Particularly, the intervention PI09 to reduce the impact of PVW was more demanded by female than male professionals (PV = 94.0%).

The greatest discrepancy (80.8%) among the experts consulted was whether or not to consider staff meetings as PIs, because in the experience of some experts, short talks, visits or presentations did not generate behavioural changes in complex variables such as PVW [38, 39].

### Discussion

In this study, we have obtained a list of 10 hierarchical PIs that reflect the consensus of experts regarding the most relevant factors that PIs aimed at preventing PVW should address. In our final list of 10 PIs, the consensus range was between 24.8% and 50.5%.

According to our results there was consensus that the starting point for preventing PVW in the country's healthcare institutions should be complemented by PIs based on emotional support, and the solidarity of the interprofessional and administrative team (ESS). This support and solidarity were considered even more valuable than institutional support. In this respect, there is evidence from Canadian nurses that the emotional support they received from their colleagues in the service provided them with greater security in the workplace [4]. As long as IP is applied independently, even if it is widely promoted, there is little likelihood of reducing long-term healthcare PVW [1]. In contrast to more integrated preventive measures that achieve a short-term reduction in both external and internal PVW [19].

There is also a consensus to apply PIs based on "externally mediated interpersonal conflict resolution" as the third most important intervention, naturally complemented by "ESS-based PIs". Likewise, the results indicate as a priority to raise the visibility of PVW in order to reduce incidences of it, and the need to incorporate PIs aimed at improving the assertive and empathic skills of those involved. For this group of multicomponent PIs, there is evidence that their application reduces PVW in the short term and can generate safety in the medium term in various health services in Australia, Canada, and the USA [1, 4, 25, 27, 28]. But it differs with the reports of Llor et al. in Spain and Kang in Korea [26, 29] because the interventions were oriented towards mental health and emergency services. It also differs with the reports of Al-ali et al. for Jordan and Hemati for Iran [32, 33] because the interventions were more oriented towards nursing staff; and in the USA because the intervention was targeted at emergency department officials [30].

Regarding the sixth PIs, which makes it possible to reduce PVW by applying preventive awareness programmes through training workshops and social activities to improve interpersonal relations between workers and civil servants, they agree with the reports of Archana et al. and Layne et al. in the USA [27, 28] when "Brainwriting techniques" are applied, which make it possible to improve interpersonal conflict resolution and consequently reduce PVW in the short term.

In this study we also find consensus on the importance of immediate punitive action for PVW. Immediacy is known to contribute to the reduction of PVW because it has a deterrent effect on perpetrators and a supportive effect on staff [2, 8]. Our results are consistent with the reports of several authors [1, 8, 21, 22] who promote prevention through punitive interventions, complemented by raising the visibility of cases of PVW [1, 8, 25]. On the need to apply recuperative PIs based on psychological accompaniment in victims of PVW complemented by PIs based on the ESS and with greater intensity in female professionals, due to its results of emotional improvement and lower job turnover, they agree with Yosep et al. from Indonesia [34] and Al-ali et al. from Jordan [32] but were more oriented for nurses from different services [32, 34]. In this regard, there must be control of organisational leadership change and staff turnover [1].

The main strength of our study is that the results were obtained by the consensus of experts, which allowed us to relate the desirable characteristics of intervention strategies with PIs that have already been applied and are therefore evidence-based. To our knowledge, this is the first consensus study to prevent PVW in health institutions in the three geographic regions of Peru.

Our results can be applied immediately as a frame of reference to choose PIs aimed at promoting interprofessional integration, formulating policies for the prevention of PVW and improving the sustainable adherence of PIs in health institutions. Initiatives that promote changes in cultural norms for gender equity in occupational health with an emphasis on preventive interventions for groups at risk of PVW such as young nurses in emergency and mental health services are pertinent.

### Conclusion

According to the consensus, the prevention of PVW should start with emotional support and solidarity among workers and have institutional support. The experts concluded that preventive PIs should be applied by integrating them with other interventions, for example, PIs based on the resolution of interpersonal conflicts, programs to raise awareness of PVW, the development of communication skills, the application of sanctions for perpetrators and the improvement of interprofessional relationships. The presence of security personnel in work environments can also aid in the prevention of PVW.

### Limitations

The number of participants per group of analysis its no proporcional; however, the participants are distributed in the three geographical regions of the country, and the minimum number per group is higher than that indicated by Bloor et al. [35]. There was natural loss of participants due to the long Delphi analysis time [31]. As a contextual preventive study influenced by environmental and cultural factors [1] the prioritised interventions are more oriented towards the Peruvian health sector.

### Supplementary Information

**Additional file 1. **Proposals for psychosocial interventions to prevent psychological violence in healthcare work. Period 2015 to 2023.

**Additional file 2. **Distribution of participants by Delphi analysis group.

## Data Availability

The dataset used and analysed during the study is available through the following link [https://figshare.com/articles/dataset/_strong_Additional_file_1_strong_Data_set_on_ranking_of_Psychosocial_interventions_at_workplace_PIW_and_socio-occupational_variables_Health_centres_in_Peru_2021-2023/23255270/1]. The data can also be obtained from the authors of this article [29]. The dataset ranking the 21 psychosocial interventions in the workplace (IPT) and socio-occupational variables is organized in a matrix of 52 × 430 datapoints corresponding to 428 people corresponding to 25 Delphi analysis groups in 66 health centres in Peru, 2021–2023.

## Abbreviations

PVW: Psychological violence in the workplace
PI: Psychosocial intervention
ILO: International Labour Organization
WHO: World Health Organization

## References

[1] Schindeler E, Reynald D (2017). What is the evidence? Preventing psychological violence in the workplace. Aggress Violent Behav.

[2] Gillespie GL, Gates DM, Fisher BS (2015). Individual, relationship, workplace, and societal recommendations for addressing healthcare workplace violence. Work.

[3] Volz NB, Fringer R, Walters B, Kowalenko T (2017). Prevalence of horizontal violence among emergency attending physicians, residents, and physician assistants. West J Emerg Med.

[4] Nowrouzi B, Rachelle I, Chai E, Usuba K, Chen A (2019). Antecedent factors in different types of workplace violence against nurses: a systematic review. Aggress Violent Behav.

[5] Hasan MI, Hassan MZ, Islam MM, Joarder T, Jobayer M (2018). Iceberg of workplace violence in health sector of Bangladesh. BMC Res Notes.

[6] Vévoda J, Vévodová Š, Nakládalová M (2018). Psychosocial risks in healthcare. Cas Lek Cesk.

[7] Mento C, Catena M, Bruno A, Muscatello MR, Cedro C, Pandolfo G (2020). Workplace violence against healthcare professionals: a systematic review. Aggress Violent Behav.

[8] Havaei F, Olvera OL, MacPhee M (2020). The impact of workplace violence on medical-surgical nurses' health outcome: a moderated mediation model of work environment conditions and burnout using secondary data. Int J Nurs Stud.

[9] Peng L, Xing K, Qiao H, Fang H, Ma H, Jiao M (2018). Psychological violence against general practitioners and nurses in Chinese township hospitals: incidence and implications. Health Qual Life Outcome.

[10] Arnetz J, Hamblin LE, Sudan S, Arnetz B (2018). Organizational determinants of workplace violence against hospital workers. J Occup Environ Med.

[11] Travetto C, Daciuk N, Fernández S, Ortiz P, Mastandueno R, Prats M (2015). Agresiones hacia profesionales en el ámbito de la salud. Rev Panam Salud Publica..

[12] Ansoleaga E, Gómez-Rubio C, Mauro A (2015). Violencia laboral en América Latina: una revisión de la evidencia científica. Vertex Rev Arg Psiquiat..

[13] Palma A, Ansoleaga E, Ahumadac M (2018). Violencia laboral en trabajadores del sector salud: revisión sistemática. Rev Med Chile..

[14] Acosta M, Parra L, Restrepo JI, Pozos BE, Aguilera ML, Torres TM (2017). Condiciones psicosociales, violencia y salud mental en docentes de medicina y enfermería. Salud Uninorte..

[15] Ministerio de Salud. Información de recursos humanos en el sector salud. Lima: MINSA; 2022. Spanish. https://cdn.www.gob.pe/uploads/document/file/3281380/Informaci%C3%B3n%20de%20Recursos%20Humanos%20en%20el%20sector%20Salud.pdf?v=1655762418.

[16] Tuya-Figueroa X, Mezones-Holguín E (2012). Violencia contra médicos: un problema por considerar en la investigación de recursos humanos en salud. Rev Peru Med Exp Salud Publica..

[17] Fitzgerald J, Schutt-Aine J, Houghton N, De Bortoli SE, Báscolo E, Alarcón G (2023). La importancia del enfoque de género en la construcción de sistemas de salud resilientes, equitativos y universales. Pan Am J Public Health..

[18] González AC, Coates A, Diaz Garcia V, Wolfenzon D (2021). Gender equality and health equity: strategic lessons from country experiences of gender mainstreaming in health. Rev Panam Salud Publica.

[19] Mohd H, Ahmad H, Ismail H, Reffin N, Chan D, Kusnin F (2023). Contemporary evidence of workplace violence against the primary healthcare workforce worldwide: a systematic review. Hum Resour Health.

[20] Organización Internacional del Trabajo, Consejo Internacional de Enfermeras, Organización Mundial de la Salud. Directrices marco para afrontar la violencia laboral en el sector de la salud. Ginebra: OIT. 2002. Spanish. https://apps.who.int/iris/bitstream/handle/10665/44072/9223134463_spa.pdf;jsessionid=6DA730621E1FFA3100F3553BDD1EDA6F?sequence=1.

[21] Spelten E, Thomas B, O’Meara P, Maguire B, Fitz D, Begg S (2020). Organisational interventions for preventing and minimising aggression directed towards healthcare workers by patients and patient advocates. Cochrane Database Syst Rev..

[22] Abeyta S, Welsh B (2022). Effects of prevention interventions on violence in the workplace: a systematic review and meta-analysis. Aggress Violent Behav.

[23] Mink J, Mitzkat A, Scharzbeck V, Mihaljevic A, Trierweiler-Hauke B, Götsch B (2022). Interprofessionelle sozialisation und zusammenarbeit auf einerinterprofessionellen ausbildungsstation-eine rekonstruktive analyse. Z Evid Fortbild Qual Gesundhwes.

[24] Raveel A, Schoenmakers B (2019). Interventions to prevent aggression against doctors: a systematic review. BMJ Open.

[25] Morphet J, Griffiths D, Beattie J, Velasquez D, Innes K (2018). Prevention and management of occupational violence and aggression in healthcare: a scoping review. Collegian.

[26] Llor B, Sánchez M, Ruiz JA (2017). User violence towards nursing professionals in mental health services and emergency units. Eur J Psychol Appl Leg Context..

[27] Archana K, Siddharth S, Piyush R, Sakshi C, Tanveer K, Upendra B (2022). Interventions for workplace violence against health-care professionals: a systematic review. Work.

[28] Layne DM, Nemeth LS, Mueller M, Schaffner MJ, Stanley KM, Martin MM (2019). Negative behaviours in health care: prevalence and strategies. J Nurs Manag.

[29] Kang J, Kim JI, Yun S (2017). Effects of a cognitive rehearsal program on interpersonal relationships, workplace bullying, symptom experience, and turnover intention among nurses: a randomized controlled trial. J Korean Acad Nurs.

[30] Wong AH, Wing L, Weiss B, Gang M (2015). Coordinating a team response to behavioral emergencies in the emergency department: a simulation-enhanced interprofessional curriculum. Western J Emerg Med.

[31] Shang Z (2023). Use of Delphi in health sciences research: a narrative review. Medicine.

[32] Al-ali NM, Al Faouri I, Al-niarat TF (2016). The impact of training program on nurses’ attitudes toward workplace violence in Jordan. Appl Nurs Res.

[33] Hemati-Esmaeili M, Heshmati-Nabavi F, Pouresmail Z, Mazlom SR, Reihani H (2018). Educational and managerial policy making to reduce workplace violence against nurses: an action research study. Iran J Nurs Midwifery Res.

[34] Yosep I, Mardhiyah A, Hendrawati H, Hendrawati S (2023). Interventions for reducing negative impacts of workplace violence among health workers: a scoping review. J Multidiscip Healthc.

[35] Bloor M, Sampson H, Baker S, Dahlgren K (2015). Useful but no Oracle: reflections on the use of a Delphi group in a multi-methods policy research study. Qual Res.

[36] Reguant M, Torrado-Fonseca M (2016). El mètode Delphi. REIRE Rev Innov Recerca Educ.

[37] Abregú LF, Bravo CJ, Abregú-Arroyo SK, Dos Santos R, Galve JL. Data set on ranking of Psychosocial interventions at workplace (PIW) and socio-occupational variables. Health centres in Peru, 2021–2023. Figshare. 2023 May. 10.6084/m9.figshare.23255270.v1

[38] Huibers M, Beurskens A, Bleijenberg G, Van Schayck CP (2007). Psychosocial interventions by general practitioners. Cochrane Database Syst Rev..

[39] Abregú-Tueros LF (2020). A systematic review of the preventive practices for psychosocial risks in Ibero-American health centers. Medwave..

