# Supplementary material for: Consensus on relevant psychosocial interventions applied in health institutions to prevent psychological violence at work: Delphi method
Source: BMC Res Notes. 2024 Jan 5;17:19. doi: 10.1186/s13104-023-06680-w (PMC10768250; doi:10.1186/s13104-023-06680-w)
Supplement: Supplementary file 1 — Additional file 1. Proposals for psychosocial interventions to prevent psychological violence in healthcare work. Period 2015 to 2023. [file 13104_2023_6680_MOESM1_ESM.docx]

**Additional file 1. Proposals for psychosocial interventions to prevent psychological violence in healthcare work. Period 2015 to 2023**

| **Authors, Year [Reference]** | **Country** | **Type of workplace violence** | | **Type of intervention** | **Health-care professionals / Effects** |
| --- | --- | --- | --- | --- | --- |
|  |  | **External ^1^** | **Internal ^2^** |  |  |
| Archana et al., 2022 [27]  Layne et al., 2019 [28] | USA  USA | X | X  X | -Workshop based training  -Brainwriting techniques | -Applicable for healthcare and administrative staff  -Generates safety in the workplace. |
| Hemati-Esmaeili et al., 2018 [33] | USA  Iran | X | X | -Prevention of violence in the emergency plan: domains such as anger, stress management and conflict resolution | -Applicable for emergency nurses with work experience.  -Significantly reduces fear |
| Kang et al., 2017 [29] | South Korea |  | X | -Cognitive rehearsal program: bullying episodes | -Applicable for nurses  -Improves interpersonal relationships and decreases job turnover. |
| Wong et al., 2015 [30] | USA |  | X | -Case simulation in interprofessional teams. | -Applicable for emergency department staff members. |
| Yosep et al., 2023 [34]  Al-ali et al., 2016 [32] | Indonesia  Jordan | X | X  X | -Remedial intervention to reduce negative impacts.  -Counselling for improving assertive and empathic communication  -Counselling in the search for positive solutions  -Training program | -Applicable for nurses.  -Reduce workplace violence among nurses |
| Glass et al. (2017) | USA |  | X | -Computer-based training (CBT) intervention | -Applicable for female homecare workers  -Reduce workplace violence |
| Kang et al., 2017 [29] | Korea |  | X | -Cognitive rehearsal program | -Applicable for nurses  -Reduce workplace bullying |
| Nowrouzi et al., 2019 [4] | Canada |  | X | -Promotion of organisational and governmental preventive policies | -Applicable for healthcare professionals  -Reduces psychological violence |
| Morphet et al., 2018 [25] | Australia |  | X | -Visibilisation of psychological violence at work (PVW) through group information and integration with other interventions. | -Applicable to healthcare professionals  -Reduces PVW |
|  |  |  |  | -Promoting "zero tolerance" policies | -Low evidence for reducing PVW |
| Acosta et al., 2017 [14] | Colombia |  | X | -Generating organisational change by integrating with resilience building | -Applicable for doctors and nurses  -Reduces PVW |
| Llor et al., 2017 [26] | Spain |  | X | -Standardised training in communication skills and interpersonal conflict management | -Applicable for mental health nurses  -Reduces PVW |
| Havaei et al., 2020 [8] | Canada | X | X | -Information system on working conditions and burnout | -Applicable for nurses |
| Raveel & Schoenmakers, 2019 [24] | Belgium | X | X | -Integrated violence prevention programme  -PVW reduction policies | -Applicable for doctors  -Moderate reduction of PVW |
| Schindeler & Reynald, 2017 [1] | Australia |  | X | -Legal support for reporting to authorities or civil servants  -Promotion of the visibility of PVW  -Training in assertive and empathetic communication skills  -Adequate and timely handling of interpersonal conflicts by officials | -Applicable for interprofessional healthcare groups  -Reduces PVW in the short |

^1^ Physical or psychological workplace violence (Type I; Type II).

^2^ Psychological workplace violence (Type III: horizontal or vertical violence).
