# Supplementary material for: Consensus on relevant psychosocial interventions applied in health institutions to prevent psychological violence at work: Delphi method
Source: BMC Res Notes. 2024 Jan 5;17:19. doi: 10.1186/s13104-023-06680-w (PMC10768250; doi:10.1186/s13104-023-06680-w)
Supplement: Supplementary file 2 — Additional file 2. Distribution of participants by Delphi analysis group. [file 13104_2023_6680_MOESM2_ESM.docx]

Additional file 2. Distribution of participants by Delphi analysis group.

| Group | | Location | | | | Number of participants | % | Chi-square test (X^2^) |
| --- | --- | --- | --- | --- | --- | --- | --- | --- |
| (Code) | | Department | Region | | |  |  |  |
| 01 | 02A | Apurímac | Sierra/Jungle | | | 9 | 2.1 |  |
| 02 | 05A | El Callao | Coastal | | | 7 | 1.6 |  |
| 03 | 08A | Huánuco | Jungle | | | 32 | 7.5 | N = 105  df = 2, p= 0.819 |
| 04 | 08B | Huánuco | Jungle | | | 36 | 8.4 |  |
| 05 | 08C | Huánuco | Jungle | | | 37 | 8.6 |  |
| 06 | 08D | Huánuco | Sierra | | | 26 | 6.1 | N = 51  df = 2; p=0.019 |
| 07 | 08E | Huánuco | Sierra | | | 15 | 3.5 |  |
| 08 | 08F | Huánuco | Sierra | | | 10 | 2.3 |  |
| 09 | 10A | Junín | Jungle | | | 21 | 4.9 |  |
| 10 | 10B | Junín | Sierra | | | 7 | 1.6 |  |
| 11 | 11A | Lima | Coastal/Sierra | | | 26 | 6.1 |  |
| 12 | 11B | Lima | Coastal | | | 26 | 6.1 | N = 110  df = 6; p=0.001 |
| 13 | 11C | Lima | Coastal | | | 12 | 2.8 |  |
| 14 | 11D | Lima | Coastal | | | 11 | 2.6 |  |
| 15 | 11E | Lima | Coastal | | | 6 | 1.4 |  |
| 16 | 11F | Lima | Coastal | | | 20 | 4.7 |  |
| 17 | 11G | Lima | Coastal | | | 25 | 5.8 |  |
| 18 | 11H | Lima | Coastal | | | 10 | 2.3 |  |
| 19 | 13A | Pasco | Sierra/Jungle | | | 9 | 2.1 |  |
| 20 | 14A | San Martín | Jungle | | | 27 | 6.3 | N = 70  df = 4; p=0.001 |
| 21 | 14B | San Martín | Jungle | | 15 | | 3.5 |  |
| 22 | 14C | San Martín | Jungle | | 15 | | 3.5 |  |
| 23 | 14D | San Martín | Jungle | | 7 | | 1.6 |  |
| 24 | 14E | San Martín | Jungle | | 6 | | 1.4 |  |
| 25 | 16A | Ucayali | Jungle | | 13 | | 3.0 |  |
| Total | |  | |  | 428 | | 100.0 |  |

Note: Participants: Minimum 6; maximum 37.
